# Biophysical dissection of the antigen-antibody interaction of the broadly reactive anti-V3 human mAb 447-52D

**DOI:** 10.1186/1742-4690-9-S2-P67

**Published:** 2012-09-13

**Authors:** A Killikelly, H Zhang, B Spurrier, C Williams, MK Gorny, S Zolla-Pazner, X Kong

**Affiliations:** 1 New York University Medical Center, New York, NY, USA; 2 Veterans Affairs New York Harbor Healthcare System, New York, NY, USA

## Background

The immunogenic third variable region (V3) of HIV-1 gp120 is a target for AIDS vaccines. V3 is recognized by mAb 447-52D, known for its ability to neutralize viruses with a GPGR beta turn motif at the apex of V3, which is characteristic of clade B viruses. Interestingly, 447-52D can also bind non-clade B V3 peptides containing a GPGQ motif. A detailed biochemical and biophysical dissection of the antigen-antibody interaction of 447-52D was undertaken to understand this disparity.

## Methods

We cloned and produced large amounts of the Fv fragment of 447-52D and a panel of mutations. We then measured their epitope binding characteristics by Isothermal Titration Calorimetry (ITC).

## Results

We assessed the Fv-V3 binding by ITC for the following mutations in residues of the mAb that are thought to mediate three key interactions: (i) Y^H100j^ of the heavy chain (H) to T (Y^H100j^T) or Y^H33^A. These two aromatic residues form a pi-cation interaction, sandwiching the side chain of R^315^ of the GPGR motif in the V3-peptide. These mutations reduce binding affinity by 56 and 171-fold, respectively. (ii) W^L91^ of the light chain (L) to A (W^L91^A). This residue packs against P^313^ of the V3 GPGR turn. This mutation reduces binding 230-fold. (iii) D^H95^R of the heavy chain or R^315^Q of the epitope. These two residues form a salt bridge between the antigen and the antibody. These mutations reduce binding by 224 and 171-fold, respectively. These data suggest a hierarchy of interactions and the salt bridge plays an important role in the affinity.

## Conclusion

mAb 447-52D binds non-clade B peptides with the R315Q variation with much less affinity, explaining why it cannot neutralize non-clade B viruses. Through probing specific contributions of individual residues by mutagenesis and ITC, we were able to fully characterize the interactions between V3 and 447-52D.

